# Concomitant Inhibition of Cytoprotective Autophagy Augments the Efficacy of Withaferin A in Hepatocellular Carcinoma

**DOI:** 10.3390/cancers11040453

**Published:** 2019-03-30

**Authors:** Sumit Siddharth, Nethaji Muniraj, Neeraj K. Saxena, Dipali Sharma

**Affiliations:** 1 Department of Oncology, School of Medicine and the Sidney Kimmel Comprehensive Cancer Center, Johns Hopkins University, Baltimore, MD 21231, USA; ssiddha2@jhmi.edu (S.S.); nmunira1@jhmi.edu (N.M.); 2 Early Detection Research Group, 22 National Cancer Institute, Rockville, MD 20892, USA; neeraj.saxena@nih.gov

**Keywords:** hepatocellular carcinoma, LC3B, cytoprotective autophagy, cathepsin-D, withaferin A

## Abstract

Hepatocellular carcinoma (HCC) is the third most common cause of cancer-related mortality, and despite recent advances in early diagnosis and therapeutics, HCC related morbidity and mortality rate continue to rise. Clearly, it is imperative to develop novel effective therapies for HCC to improve long-term survival of HCC patients. We found that Withaferin A (WFA), a bioactive compound derived from *Withania somnifera*, is an effective agent for HCC inhibition. Interestingly, we observed that in addition to inducing apoptotic cell death, WFA also induces autophagy in HCC cells. Utilizing mRFP-EGFP-LC3B, LC3B-GFP/Lysotracker and LC3B-GFP/Rab7-RFP, we show that WFA induces autophagosomes-lysosomes fusion. WFA-induced autolysosomes exhibit intact protein degradation activity as evident with cathepsin-D activation and DQ-BSA assays. Importantly, we present that inhibiting WFA-induced autophagy either by blocking autophagosome-formation or by elevating lysosomal pH (Chloroquine and Bafilomycin) enhances WFA-induced growth-inhibition and apoptosis, indicating the presence of cytoprotective autophagy. Indeed, WFA and CQ combination shows synergism and higher efficacy in comparison to either monotherapy. Collectively, we reveal that the efficacy of WFA is somewhat diminished by the concomitant induction of cytoprotective autophagy which can be successfully conquered by cotreatment with CQ, and we pave the way for development of a novel combination therapeutic strategy for HCC.

## 1. Introduction

Hepatocellular carcinoma ranks as the sixth most common cancer and the third major cause of cancer related death, with an age-adjusted global incidence of 10.1 cases per 100,000 persons/year [1]. Annual percent change (APC) in liver cancer and invasive intrahepatic bile duct cases increased from 0.2 in 1975 to 1.8 in 2015. Globally, more than half a million people are diagnosed with hepatocellular carcinoma every year, with approximately 20,000 new cases in USA alone. An estimate by GLOBOCAN revealed approximately 782,000 new cases of hepatocellular carcinoma and 745,000 liver cancer related deaths in 2012 globally. According to the World Health Organization (WHO) hepatocellular carcinoma remains a prominent cause of cancer related mortality across the globe [2]. The average overall survival rates for liver cancer fluctuates between six to 20 months, which has made hepatocellular carcinoma the most aggressive primary hepatic malignancy [3]. The therapeutic options for hepatocellular carcinoma are challenging due to its complex pathophysiology. Sorafenib, an oral tyrosine kinase inhibitor, is used as an exclusive targeted treatment strategy against hepatocellular carcinoma, and has been shown to reduce the mortality burden to some extent [4]. Reports reveal that sorafenib increases median survival for only three months compared to placebo in patients with advanced hepatocellular carcinoma [5,6]. Meta-analysis and multicenter clinical trial studies showed that sorafenib prolongs survival and delays disease progression, but is accompanied with multiple side effects like diarrhea, fatigue, hypertension and dermatologic toxicity [7]. Genetic heterogeneity of hepatocellular carcinoma remains a challenge in the search of novel therapeutics. Clearly, it is imperative to develop new effective treatment strategies for HCC to improve long-term survival of HCC patients.

Bioactive compounds play a significant role in drug discovery [8,9,10,11], mainly in the field of cancer, as is evident by the fact that most of the FDA approved drugs (Vincristine (*Cathranthus roseus*), vinblastine (*Cathranthus roseus*), cytarabine (*Tectitethya crypta*), doxorubicin (Sterptomyces peucetius), etoposide (*Podophyllum peltatum*), paclitaxel and docetaxel (*Taxus baccata*)) have natural product origins [12,13]. There is a wide interest in investigating bioactive compounds derived from medicinal plants that have been used in traditional medicine for various ailments for centuries. *Withania somnifera*, also known as Ashwagandha or Indian winter cherry, is one such medicinal plant whose root extract has shown pharmacological properties and efficacy [14,15,16]. Investigations aiming to isolate the bioactive compounds from the root extract of *Withania somnifera* led to the discovery of 14 bioactive compounds known as Withanolides [17,18]. Withaferin A (WFA) being the most abundant bioactive compound among all Withanolides, garnered much interest among research groups, and has been examined for its anticancer potential in various cancers [19]. WFA exhibits therapeutic potential against cardiac ischemia reperfusion injury [20]. WFA has been shown to inhibit growth and induce apoptosis in colon cancer [21], colorectal cancer [22], breast cancer [23], and oral cancer [24]. Acting as a potential c-Met inhibitor, WFA inhibits stemness in pancreatic cancer [25] and also inhibits epithelial-mesenchymal transition in non-small cell lung cancer cells [26]. Phosphokinase array analyses revealed that WFA activates ERK/RSK axis and mediates growth inhibition via death receptor 5 (DR5) upregulation [27,28]. Withania somnifera extract is being investigated for its efficacy for anxiety, schizophrenia and bipolar disorder in clinic (ClinicalTrials.gov Identifier: NCT00761761, NCT01311180, NCT01793935) and has been reported to have clinical benefits for schizophrenia [29].

The present study is designed to systematically investigate anti-HCC efficacy of WFA focusing on the functional involvement of autophagy. We report that while WFA treatment results in growth-inhibition and apoptotic-induction in HCC, it also induces autophagic process manifested with increase in lipidated LC3B, activation of various ATG proteins, formation of autophagolysosomes, and efficient proteolytic activity in WFA-treated HCC cells. Importantly, we show that WFA-induced autophagy is cytoprotective at the functional level and a therapeutic strategy combining WFA and autophagy inhibitors is highly effective in HCC.

## 2. Results

### 2.1. Withaferin a Treatment Inhibits Viability and Clonogenicity of Hepatocellular Carcinoma Cells

**Figure 1:**
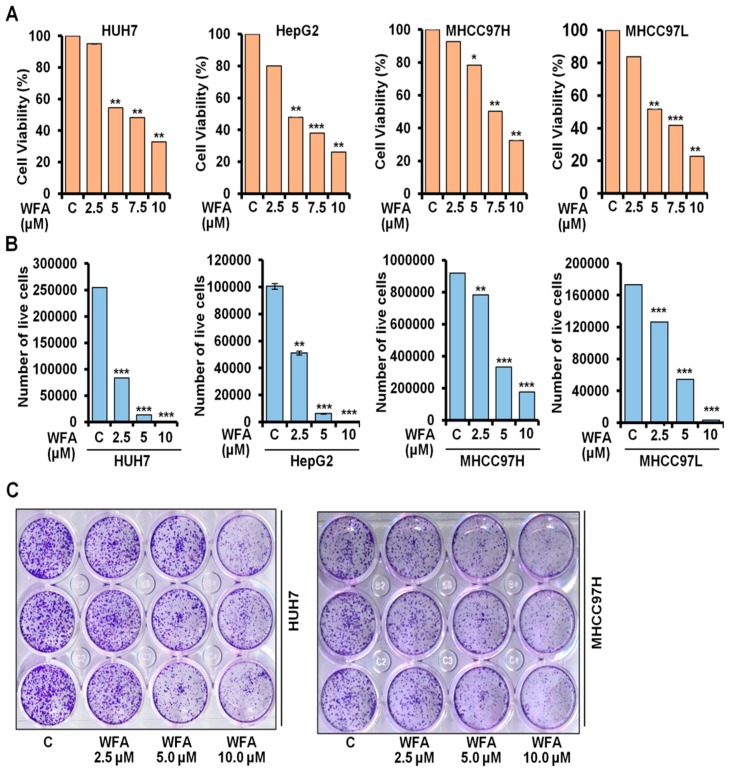
Withaferin A inhibits growth and clonogenicity of HCC cells. (**A**) Cell viability of Huh7, HepG2, MHCC97H and MHCC97L cells was examined using MTT assay after treatment with indicated concentrations of WFA compared to respective vehicle treated controls (denoted by “C”). (**B**) Huh7, HepG2, MHCC97H and MHCC97L cells were treated with mentioned concentrations of WFA followed by trypan blue dye exclusion assay. (**C**) Huh7 and MHCC97H cells were treated with indicated concentrations of WFA and subjected to clonogenicity assay. * *p* < 0.05, compared with control; ** *p* < 0.01, compared with control; *** *p* < 0.001, compared with control.

Previous study from our lab showed the growth-inhibitory and pro-apoptotic effects of WFA in HepG2 and Huh7 cells [28]. Evaluation of anti-proliferative efficacy of WFA on multiple HCC cells showed that WFA treatment significantly decreased the anchorage-dependent cell viability of multiple HCC cells in a dose-dependent manner, with an IC50 of 5 µM in Huh7, HepG2 and MHCC97L cells; and 7.5 µM in MHCC97H cells, respectively (Figure 1A). Trypan blue dye exclusion assay revealed a five-fold and two-fold reduction in Huh7 and HepG2 viable cells, respectively, with 2.5 µM WFA treatment (Figure 1B). However, more than a three-fold reduction in MHCC97H and MHCC97L viable cells was observed with 5 µM WFA treatment (Figure 1B). To thrive under unfavorable conditions, cancer cells possess clonogenic potential, which aids their survival as colonies of cells. To analyze the anti-colony forming ability of WFA in HCC cells, we performed a clonogenic cell survival assay upon WFA treatment. Both Huh7 and MHCC97H cells revealed more than a 70% decrease in colony-forming ability upon WFA treatment. Together, these results present the anti-proliferative and anti-clonogenic ability of WFA against HCC cells.

### 2.2. Increased Conversion of LC3B upon Withaferin Treatment Indicates Autophagic Induction

**Figure 2:**
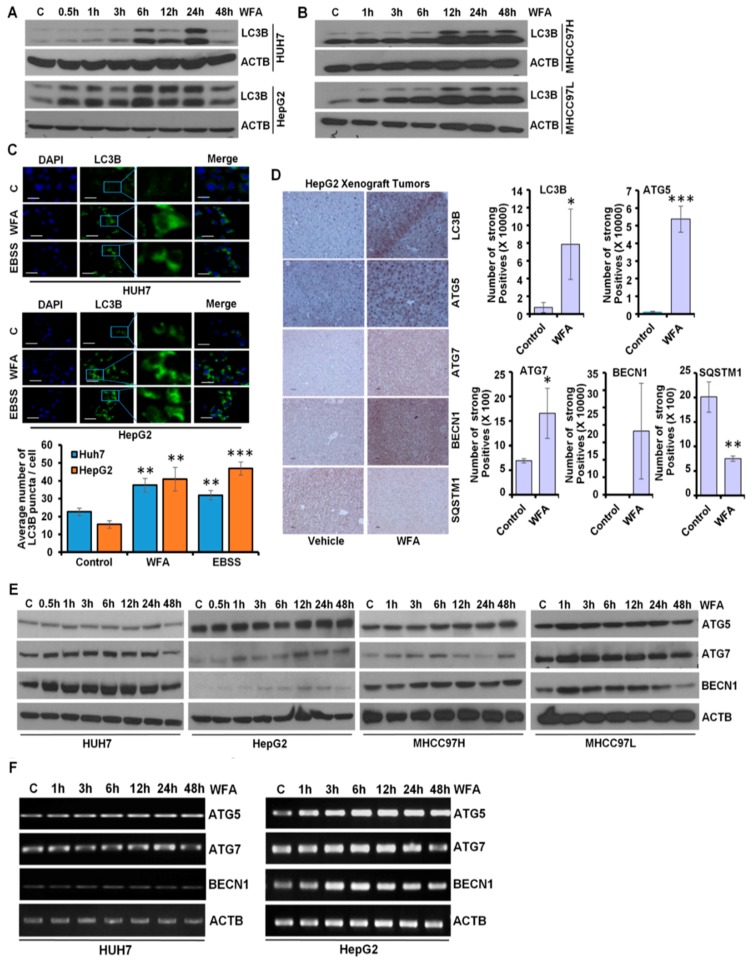
Withaferin A induces LC3B conversion and augments autophagy in vitro and in vivo. (**A**,**B**) Expression level of LC3B in HCC cells after treatment with 5 µM of WFA by immunoblotting. ACTB served as the loading control. (**C**) HCC cells were transfected with a GFP-tagged LC3B-encoding plasmid, followed by treatment with 5 µM WFA or EBSS. EBSS was used as a positive control for autophagy-induction. Representative images are shown. LC3B puncta were counted and shown as bar diagram. ** *p* < 0.01, compared with control; *** *p* < 0.001, compared with control. Scale bar 20 μm. (**D**) HepG2 derived *xenograft* tumors from vehicle-treated and WFA-treated mice were subjected to immunohistochemical analysis for autophagy related proteins (LC3B, ATG5, ATG7, BECN1 and SQSTM1). IHC signals were quantified using Aperio ImageScope Software, Leica and shown as bar graphs. * *p* < 0.05, compared with control; ** *p* < 0.01, compared with control; *** *p* < 0.001, compared with control. Scale bar 100 μm (**E**) HCC cells were treated with 5 μM WFA and immunoblotted for the expression level of autophagy related proteins (ATG5, ATG7, BECN1 and SQSTM1). ACTB served as the loading control. (**F**) Total RNA was extracted from the WFA treated and vehicle treated HCC cells and the expression of ATG5, ATG7 and BECN1 was studied. ACTB was used as the loading control.

The autophagic process, an act of “self-degradation”, impacts various aspects of HCC growth and metastatic progression, as well as modulating the therapeutic efficacy of various drugs targeting HCC [30,31], hence why we explored the involvement of autophagy in HCC cells upon WFA treatment. Autophagy related protein ATG4 cleaves microtubule-associated protein 1 light chain 3 α (MAP1LC3B/LC3B) to form LC3B-I which then conjugates with phosphatidylethanolamine to form LC3B-II. LC3B-I to LC3B-II conversion is an indicator of autophagy activity [32]. We observed a dose-dependent accumulation of LC3B-II in Huh7, HepG2, MHCC97H and MHCC97L HCC cells upon WFA treatment, which indicated the involvement of autophagy (Figure 2A,B). Huh7 and HepG2 cells exhibited an increase in lipidated LC3B till 24 h-post treatment and showed a decline in lipidated LC3B 48 h-post treatment, whereas MHCC97H and MHCC97L cells showed an increased level of lipidated LC3B till the 48 h-post treatment. A transient increase in the lipidated form of LC3B is sufficient to induce the autophagic response [33].

Immunofluorescence analyses of WFA treated HCC cells demonstrated the presence of a significantly higher number of autophagic vacuoles, marked with LC3B puncta compared to vehicle treated control cells (Figure 2C). These in vitro observations were also corroborated using in vivo HCC tumors from an HCC-xenograft assay. Indeed, immunohistochemical analysis revealed elevated expression of LC3B, ATG5, ATG7 and BECN1 with decreased expression of SQSTM1 in HepG2 xenografts from WFA-treated mice in comparison to vehicle treated mice (Figure 2D). Autophagic process is orchestrated by various autophagy-related proteins that mediate the formation of an initiation complex, phagophore initiation and elongation, autophagosome formation, and fusion of autophagosomes with lysosomes [34]. WFA treated HCC cells showed increased expression of autophagy related proteins, such as, ATG5, ATG7 and BECN1 (Figure 2E). Increased mRNA expression of ATG5, ATG7 and Beclin1 was also observed in HCC cells upon WFA treatment (Figure 2F). Collectively, these data show that WFA augments autophagy in HCC.

### 2.3. Withaferin a Augments the Formation of Autophagosomes in Hepatocellular Carcinoma Cells

**Figure 3:**
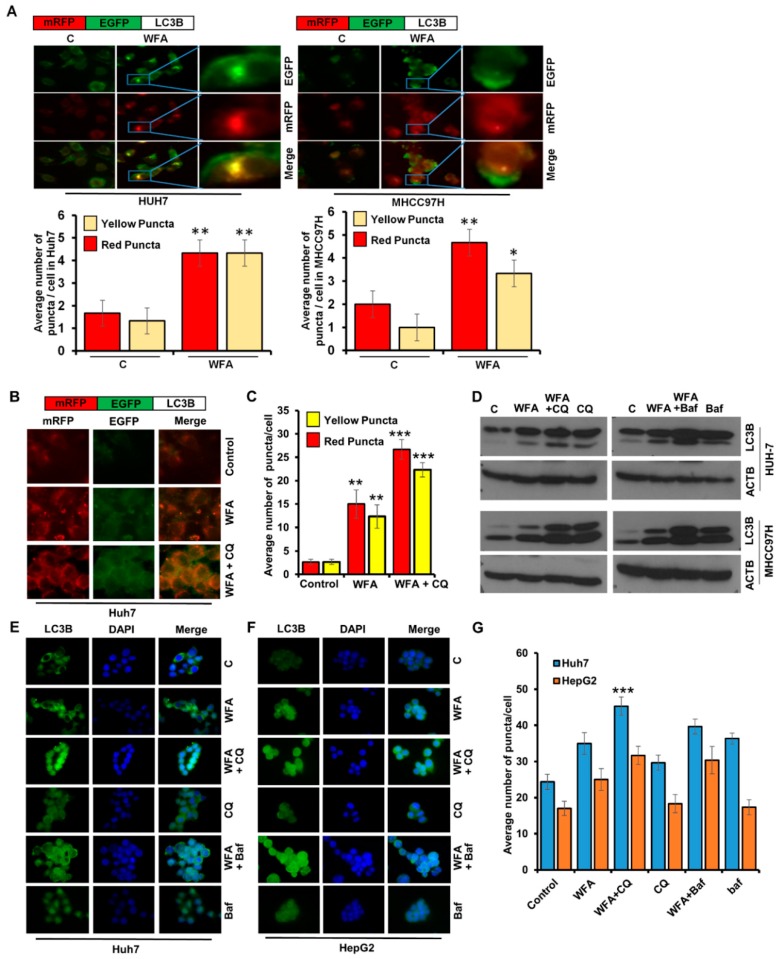
Withaferin A increases the autophagic flux in HCC cells. (**A**) Upper panel represent the schematic diagram of tfLC3B. Huh7 and MHCC97H cells were transfected with tfLC3B and treated with 5 µM WFA or EBSS. Representative images are shown. Red and yellow puncta were counted and shown as bar diagram. * *p* < 0.05, compared with control; ** *p* < 0.01, compared with control, Scale bar 10 μm. (**B**,**C**) Upper panel represent the schematic diagram of tfLC3B. Huh7 cells were transfected with tfLC3B and treated with 5 μM WFA in combination with 25 µM CQ. Representative images are shown. Red and yellow puncta were counted and shown as a bar diagram. ** *p* < 0.01, compared with control; *** *p* < 0.001, compared with control, Scale bar 10 μm (**D**) Huh7 and MHCC97H cells were treated with 5 μM WFA in combination with 25 µM CQ and 200 nM bafilomycin and subjected to immunoblot analyses for the expression level of LC3B. ACTB served as the loading control. (**E**–**G**) Huh7 and MHCC97H cells were treated with 5 μM WFA in combination with 25 µM CQ and 200 nM bafilomycin and subjected to immunofluorescence analyses for LC3B. Representative images are shown. Green puncta were counted and shown as bar diagram. *** *p* < 0.001, compared with WFA alone, Scale bar 10 μm.

It is known that increased levels of LC3B-II upon a drug treatment can be observed either due to increased synthesis of autophagosomes or due to the decreased turnover of autophagosomes because of slower trafficking to the lysosomes [35]. To examine whether WFA induces autophagosomes in HCC cells, we transfected Huh7 and MHCC97H cells with mRFP-EGFP-LC3B, a tandem fluorescent-tagged LC3B plasmid that can identify autophagosomes (GFP-positive and RFP-positive merged as yellow) and autophagolysosomes (GFP-negative and RFP-positive merged as red) [36]. WFA treated Huh7 and MHCC97H cells exhibited an increase in yellow fluorescence, which indicated increased autophagosomes formation (Figure 3A). To evaluate that WFA indeed induces autophagic flux, HCC cells transfected with mRFP-EGFP-LC3B were treated with WFA in combination with autophagic inhibitors such as chloroquine and bafilomycin A1 and colocalization of red and green puncta were examined. An increase in red and yellow puncta in cells treated with autophagic inhibitors and WFA combination in comparison to WFA alone indicated increased autophagic flux (Figure 3B,C). Also, WFA induced accumulation of lipidated LC3B was further enhanced in the presence of autophagic inhibitors as observed with immunoblotting (Figure 3D) and visualization of endogenous LC3B puncta (Figure 3E–G) in HCC cells. These results show that WFA increases autophagic flux.

**Figure 4:**
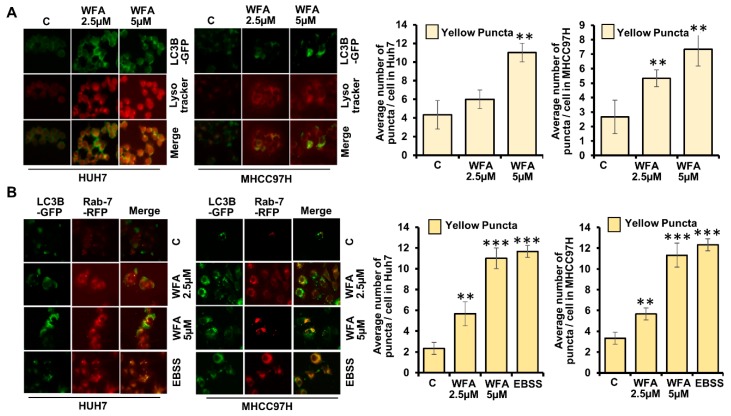
Withaferin A elevates the fusion of autophagosomes with lysosomes. (**A**) Huh7 and MHCC97H cells were transfected with GFP-LC3B encoding plasmid, treated with 5 µM of WFA or EBSS, followed by Lysotracker red staining. Representative fluorescence images are shown. Number of yellow puncta shown as bar graphs. ** *p* < 0.01, compared with control. (**B**) Huh7 and MHCC97H cells were co-transfected with GFP-LC3B and RFP-Rab7 followed by 5 μM WFA or EBSS treatment. Representative fluorescence images are shown here. Bar graphs show number of yellow puncta. ** *p* < 0.01, compared with control; *** *p* < 0.001, compared with control. Scale bar 10 μm.

Next, we investigated whether WFA treatment increases the fusion of autophagosomes with lysosomes to form autophagolysosomes in HCC cells. Since autophagosomes are decorated with LC3B and lysosomes are acidic vesicles, they can be distinguished by staining for GFP-LC3B and an acidic pH marker-LysoTracker-Red. We transfected HCC cells with GFP-LC3B, co-stained with LysoTracker-Red and observed the fusion of autophagosomes with lysosomes upon WFA treatment. WFA treated HCC cells exhibited significant overlap of GFP-LC3B signals with LysoTracker-Red signals (observed as yellow puncta) indicating the formation of autophagolysosomes in comparison to vehicle treated cells (Figure 4A). Rab7 is a member of a large superfamily of Ras-like GTPases and plays an important role in autophagosomal maturation, lysosomal biogenesis, positioning, trafficking and degradation [37]. Rab7 marks the maturation of autolysosomes and its upregulation enhances the autophagic process [37], hence, we co-transfected HCC cells with GFP-LC3B and RFP-Rab7 plasmids to detect the autophagolysosomes. Formation of autophagolysosomes was observed as a merge of autophagosomes (marked with GFP-LC3B, green signal) and lysosomes (marked with RFP-Rab7, red signal) and displayed as yellow signals (GFP positive/RFP positive) in HCC cells treated with WFA similar to EBSS-treated cells, while untreated cells showed weak red and green signals (Figure 4B). Taken together, these data present that WFA induces the formation of autophagolysosomes in HCC cells.

### 2.4. Induction of Lysosomal Activity Upon Withaferin a Treatment Exhibits a Functional Autophagic Response

**Figure 5:**
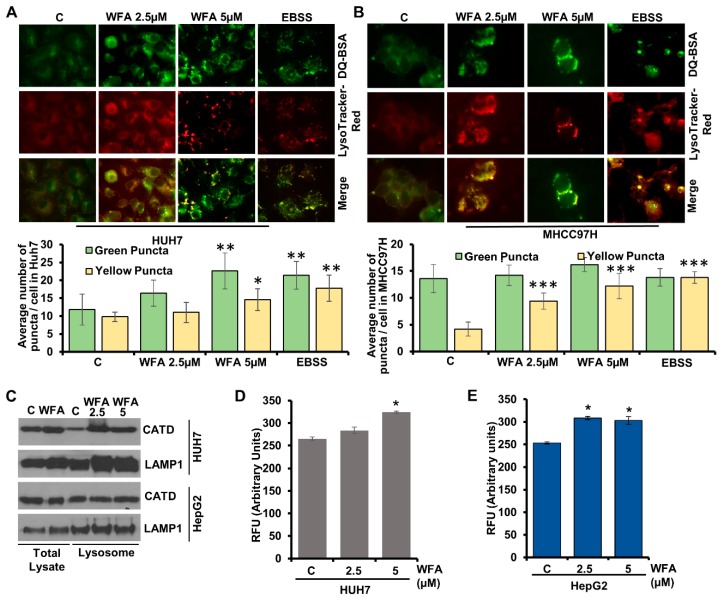
Withaferin A induces proteolytic degradation and Cathepsin D in lysosomes. (**A**,**B**) Huh7 and MHCC97H were treated with DQ-BSA followed by treatment with 5 µM of WFA or EBSS; fixed and stained with Lysotracker-Red. Representative images are shown. Bar diagrams show quantification of green and yellow puncta. * *p* < 0.05, compared with control; ** *p* < 0.01, compared with control; *** *p* < 0.001, compared with control. Scale bar 10 μm (**C**) Western blot analysis showing Cathepsin D and LAMP1 expression levels in the total cellular lysate and in the lysosomal extract of vehicle and WFA treated HUH7 and HepG2. (**D**,**E**) Cathepsin D activity assay using lysates from vehicle and WFA treated Huh7 and HepG2 cells.

A culminating step that defines autophagic response, and also separates it from other cellular trafficking and degradation machineries, is the lysosomal degradation of the cargo cytoplasmic material [33]. To confirm that WFA induced lysosomal activity in HCC cells, we utilized the DQ-BSA (BSA derivative whose green fluorescence gets quenched excluding when it is cleaved by proteolytic enzyme) assay. HCC cells incubated with DQ-BSA and LysoTracker-Red showed dequenching of DQ-BSA (green fluorescence is retained) in lysosomes (exhibiting red fluorescence) upon WFA treatment showing colocalization of red and green fluorescence (merge as yellow), similar to EBSS-treated cells whereas DQ-BSA was quenched in vehicle-treated cells (Figure 5A,B). While multiple other cellular degradation processes degrade their substrates to short peptides, autophagic degradation via lysosomal proteases uniquely degrades the cargo in autophagolysosomes to their constituting amino acids, fueling the metabolic reactions or repair processes in the cell [33]. Cathepsin-D is one of the major lysosomal enzyme required for degrading cargo in autolysosomes [38], and to confirm the involvement of Cathepsin-D, we examined Cathepsin-D expression and activity, respectively. The lysosomal extract of WFA treated HCC cells showed increased Cathepsin-D level compared to the vehicle treated control cells (Figure 5C–E). An increase in expression and activity of Cathepsin-D along with DQ-BSA-dequenching, showed that WFA treated HCC cells undergo complete autophagic response.

### 2.5. Withaferin-Induced Autophagy is Cytoprotective in Function

**Figure 6:**
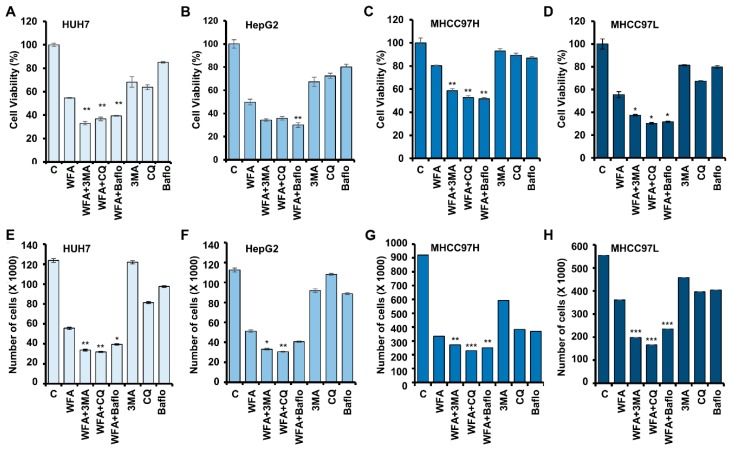
Withaferin A-mediated autophagy in HCC cells is cytoprotective in nature. (**A**–**D**) Cell viability of various HCC cells after treatment with 5 µM WFA alone and in combination with 4 mM of 3MA (3-Methyl adenine), 25 µM CQ or 200 nM bafilomycin, respectively. (**E**–**H**) HCC cells were treated with 5 µM WFA alone and in combination with 4 mM 3MA, 25 µM CQ and 200 nM bafilomycin, respectively, and subjected to trypan blue dye exclusion assay. * *p* < 0.05, compared with Withaferin A; ** *p* < 0.01, compared with WFA; *** *p* < 0.001, compared with Withaferin A.

Cells utilize cytoprotective autophagy for survival and escape from apoptotic cell death upon drug treatment [39]; hence inhibition of cytoprotective autophagy may sensitize tumor cells to therapeutic approaches. On the other hand, cytotoxic-autophagy induces the apoptotic effects of drug treatments; hence inhibition of cytotoxic-autophagy promotes cell survival [40]. To determine the functional impact of WFA-induced autophagy in HCC cells, we blocked autophagy using autophagy inhibitors, 3MA (inhibits autophagy by blocking autophagosome formation via class III PI3K inhibition), bafilomycin (inhibits autophagy by inhibiting the fusion of autophagosomes and lysosomes) or chloroquine (inhibits autophagy by elevating lysosomal pH). Interestingly, we observed that the combined treatment of WFA with 3MA, CQ or bafilomycin, respectively, enhanced the apoptotic effects in comparison to WFA alone. Approximately, >1.5 fold reduction in cell viability was noted when WFA was combined with 3MA or CQ or Bafilomycin in Huh7, HepG2, MHCC97H and MHCC97L HCC cells compared to WFA treatment alone (Figure 6A–H). Of note, 3MA or CQ or bafilomycin revealed slight changes in cell viability compared to vehicle treatment in Huh7, HepG2, MHCC97L HCC cells (Figure 6A–F,H) albeit MHCC97H cells showed a reduction in number of viable cells in trypan blue exclusion assay upon CQ and bafilomycin treatment (Figure 6G). Collectively, these findings reveal that WFA induces cytoprotective autophagy in HCC cells.

### 2.6. Simultaneous Inhibition of Cytoprotective Autophagy Along with Withaferin a Treatment Synergistically Inhibits Hepatocellular Carcinoma Cells

**Figure 7:**
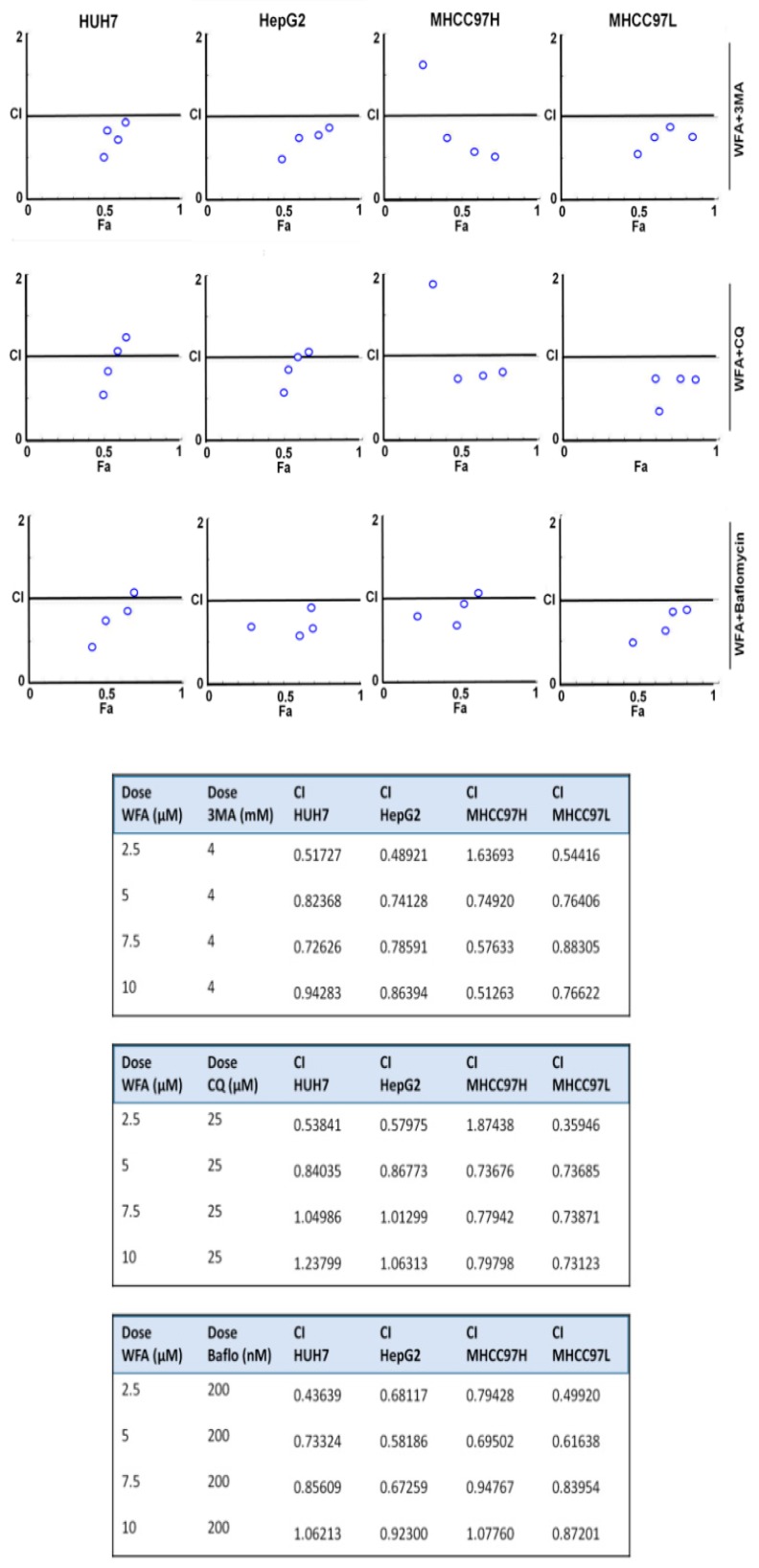
Synergistic interaction between Withaferin A and autophagic inhibitors. Inhibitory effects of WFA combined with 3MA, Chloroquine (CQ) and bafilomycin, respectively, in various HCC cells. The CI value was calculated using the Chou-Talalay method. CI < 1, CI = 1 and CI > 1 indicates synergistic, additive and antagonistic effects, respectively.

**Figure 8:**
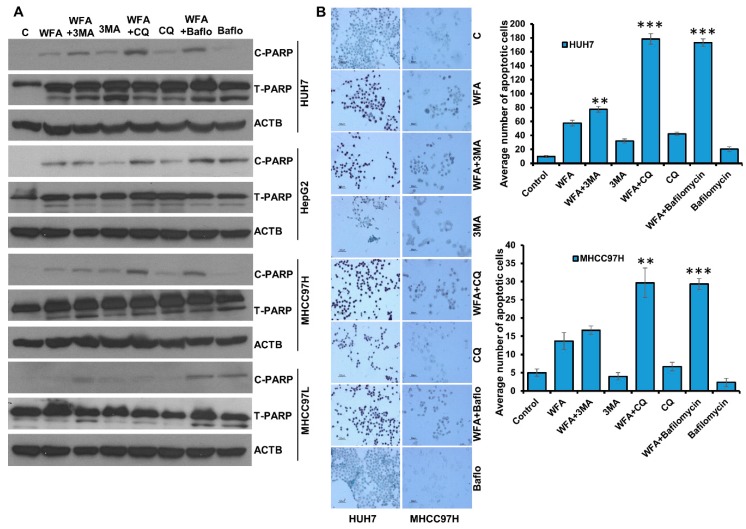
Combined treatment with Withaferin A and autophagic inhibitors induces apoptosis in HCC cells. (**A**) HCC cells were treated with 5 µM WFA alone and in combination of 4 mM 3MA, 25 µM CQ and 200 nM bafilomycin, respectively, total cellular lysates were prepared and subjected to western blot analysis to examine the expression level of Cleaved-PARP (C-PARP) and Total-PARP (T-PARP), respectively. ACTB served as the loading control. (**B**) Huh7 and MHCC97H were treated with 5 µM WFA alone or co-treated with 4 mM 3MA, 25 µM CQ and 200 nM bafilomycin, respectively, and subjected to TUNNEL assay following the manufacturer’s protocol. Images were captured microscopically at 100× (Huh7) and 50× (MHCC97H) magnification. Bar graphs show number of apoptotic cells. ** *p* < 0.01, compared with WFA; *** *p* < 0.001, compared with WFA, Scale bar, 100 μm.

To understand whether the interaction between WFA and 3MA, CQ or bafilomycin, respectively, is additive, synergistic or antagonistic in nature, we analyzed the viability data using Compusyn (Compusyn Inc., Paramus, NJ, USA) software. Dose effect analysis of WFA in combination with 3MA, CQ or Bafilomycin at various concentrations demonstrated significant synergistic interactions (Figure 7) suggesting that a combination approach including WFA and autophagic inhibitors might be more efficient than monotherapy approach. It is interesting to note that different HCC cells exhibited a slight variation in combination index. Henceforth, we investigated the efficacy of combination regimens involving WFA and autophagy inhibitors. Huh7, HepG2, MHCC97H and MHCC97L HCC cells treated with WFA and autophagy inhibitors-CQ, Bafilomycin and 3MA combinations exhibited elevated levels of cleaved PARP in comparison to cells treated with WFA alone (Figure 8A). HCC cells treated with 3MA, CQ and Bafilomycin alone did not show a substantial increase in cleaved PARP (Figure 8A). Next, the efficacy of a combination approach of WFA and autophagy inhibitors was evaluated using TUNEL assay. We observed a higher number of apoptotic nuclei in HCC cells treated with WFA and autophagy inhibitors in combination as compared to vehicle treatment (Figure 8B). Treatment with autophagic inhibitors alone did not result in substantial apoptosis. These results show that WFA treatment synergizes with autophagic inhibitors and induces a significant apoptotic response in HCC cells. Together, our data provide strong evidence that WFA inhibits HCC growth, but also induces cytoprotective autophagy that can be efficiently circumvented via combining autophagy inhibitors.

## 3. Discussion

Autophagy, a term coined by Belgian cytologist and biochemist Christian De Duve in 1963, literally means “self-eating” and is center to multiple biological processes. Although it was known that autophagy involves single- or double-membraned intracellular vesicles containing various damaged and disintegrating cytoplasmic organelles in 1960s, the genes associated with autophagy-ATG (AuTophaGy) genes were discovered much later on in 1993 [41]. This propelled the field forward, and now we know that various pharmacological agents or bioactive compounds inhibit or activate autophagy to impact multiple disease states, highlighting the physiological importance of autophagic process. In this study we present that Withaferin A (WFA) inhibits HCC growth and concomitantly induces autophagy. Characterized with two main features, namely the involvement of cytoplasmic material and culmination with lysosomal degradation, autophagy is a multistep process. We examined the impact of WFA treatment on multiple steps of autophagic process to unequivocally establish that WFA indeed induces autophagy in HCC cells. Observation that WFA treatment increased level of lipidated LC3B and LC3B-puncta in HCC cells prompted us to examine the fusion of autophagosomes and lysosomes in WFA-treated HCC cells. Multiple approaches such as microscopic evaluation of tandem GFP-RFP-tagged LC3B, colocalization of LysoTracker-Red staining with GFP-LC3B and overlap of fluorescence signals of GFP-LC3B and RFP-Rab7 were used to establish that WFA treatment increased the formation of autophagolysosomes. An important step in autophagy is the degradation of cytoplasmic cargo mediated by the lysosomal hydrolyses-cathepsin proteases. Utilizing DQ-BSA assay and direct examination of cathepsin D levels and activity, we found that WFA-treated cells possess higher lysosomal activity. Together, our data showed that WFA inhibits growth and proliferation of HCC cells as well as induces autophagy.

Autophagy plays an important role in mediating therapeutic resistance in HCC cells [31,42]. Induction of autophagy can affect cancer cells in multiple different ways and based on its functional impact, it can be characterized as cytoprotective, cytotoxic, cytostatic or nonprotective autophagy [40]. Cancer cells are generally known to protect themselves against therapeutics or environmental threats via undergoing cytoprotective autophagy whose inhibition increases the efficacy of anticancer drugs. This is essentially the basis of the development of approaches investigating autophagy-inhibition as an additive to cancer therapy. On the other hand, cytotoxic autophagy would enhance the therapeutic potential of a drug, resulting in increased inhibition of cancer cells hence as its inhibition reduces drug effectiveness [43]. We previously showed the involvement of cytotoxic autophagy in adiponectin-induced inhibition of growth and metastatic progression of breast cancer cells, and as expected, the inhibition of autophagy protected breast cancer cells against adiponectin [44]. If a drug induces cytotoxic autophagy, then a pharmacological approach to inhibit autophagy would undermine the therapeutic intent of the approach. Yet another functional form of autophagy is cytostatic, characterized by growth inhibition, reduced clonogenic survival and an association with senescence and tumor dormancy [45]. In contrast to other three functional forms of autophagy, a nonprotective autophagy is essentially evidenced when a treatment or an environmental cue results in the apparent induction of autophagic response, but its inhibition does not inhibit or sensitize a drug treatment [40]. Autophagic induction can uniquely influence the efficacy of drugs. Interestingly, we found that WFA-induced autophagy in HCC cells is cytoprotective at the functional level as inhibition of autophagy using 3MA, Bafilomycin or CQ enhanced the efficacy of WFA treatment. In fact, combination index (CI) analyses showed that WFA and autophagy inhibitors synergistically inhibited growth of HCC cells, indicating that a combination therapeutic approach involving WFA and clinically viable autophagy inhibitors might be an effective strategy against HCC. It is important to note that molecular mechanisms underlying the anti-cancer function of WFA are cancer-type specific. While HCC cells exhibited induction of cytoprotective autophagy in response to WFA treatment, breast cancer cells undergo a non-protective autophagy with impaired lysosomal activity and cell death via energetic impairment upon WFA treatment [46]. We also observed that combining WFA and 2DG (2-Deoxyglucose) is a useful strategy for targeting breast cancer [46]. These observations support a careful mechanistic investigation prior to designing combination therapeutic regimens using WFA for different cancer types. 

Inhibition of autophagy using various strategies have shown benefits for reducing growth and metastatic progression of HCC cells. Induction of autophagy in HCC cells increases fluid shear stress-induced migration and invasion that can be successfully inhibited with 3MA and autophagic inhibition by silencing ATG5 [47]. Nitric oxide disrupts Beclin1/Vps34 association and inhibits autophagy resulting in increased apoptosis in HCC cells [48]. Combination therapy of chloroquine and doxorubicin exhibits increased anti-tumor activity in HCC models [49]. In addition to chemical inhibitors of autophagy, few bioactive strategies including red ginseng extract have been shown to inhibit cytoprotective autophagy and sensitize HCC cells to therapy [50]. Interestingly, sorafenib induces autophagy in HCC cells compromising its efficacy but wogonin, (5,7-dihydroxy-8-methoxyflavone), a flavonoid derived from the root of the medicinal herb *Scutellaria baicalensis*, effectively inhibits sorafenib-induced autophagy and combining sorafenib with wogonin yields better efficacy [51]. Bioactive compound Agrocybe aegerita lectin (AAL), a lectin isolated from the fungus Agrocybe aegerita, has shown that the induction of cytoprotective autophagy can be alleviated with CQ treatment leading to synergistic inhibition of HCC growth [52]. Combining autophagy inhibitors with Meloxicam, a selective cyclooxygenase-2 (COX-2) inhibitor enhances HCC inhibition [53]. Nanodiamond autophagy inhibitor has also been shown to improve cancer therapy [54]. Our results that combined WFA with autophagy inhibitors showed enhanced efficacy in HCC, and they are in line with previous studies where drug-induced cytoprotective autophagy is successfully abrogated with combination approaches.

Hydroxychloroquine (HCQ) has been used in multiple clinical trials to inhibit cytoprotective autophagy and enhance drug effectiveness. A multicenter phase I/II trial of everolimus (10 mg daily) and HCQ determined maximum tolerated dose of HCQ with everolimus and estimated the six-month progression-free-survival (PFS) in renal cell carcinoma patients. These patients did show stable disease and partial response in 67% patients, and HCQ was deemed an acceptable autophagy inhibitor for RCC patients [55]. Combining HCQ (600 mg oral, daily) with histone deacetylase inhibitor Vorinostat exhibited improved antitumor immunity in metastatic colorectal cancer patients [56]. Various combination strategies are evaluating the efficacy of HCQ with paclitaxel and carboplatin in advanced/recurrent non-small cell lung cancer (ClinicalTrials.gov-NCT01649947); HCQ with Capecitabine, Oxaliplatin and Bevacizumab in metastatic colorectal cancer (ClinicalTrials.gov-NCT01006369; and HCQ with Sorafenib in solid tumors (ClinicalTrials.gov-NCT01634893). Several studies have evaluated, or are evaluating the effects of CQ in various combinations, for example, a phase II trial of myeloma patients examining the combination of CQ with Velcade and Cyclophosphamide (ClinicalTrials.gov-NCT01438177); CQ with taxane for advanced metastatic breast cancer (ClinicalTrials.gov-NCT01446016). Multiple studies evaluating CQ/HCQ combinations with many established anti-cancer therapies are listed in ClinicalTrials.gov, indicating heightened interest in research community regarding autophagy inhibitors. 

## 4. Materials and Methods

### 4.1. Cell Culture and Reagents

Hepatocellular carcinoma cells (Huh7, HepG2, MHCC97H and MHCC97L) were grown and cultured in DMEM (Corning, cellgro, Manassas, VA, USA; cat #10-013-CV) supplemented with 10% FBS and 1% antibiotic-antimycotic. MHCC97H and MHCC97L cells represent cells that exhibit similar malignancy but differ in metastatic potential representing high vs. low lung metastasis. All the cell lines are cultured and stored following instructions from the supplier and frozen stocks are used within low passage number. Withaferin A (WFA) was procured from Calbiochem EMD Millipore (Billerica, MA, USA). MAP1LC3B/LC3B (3868), ATG5 (12994), ATG7 (8558), BECN1 (3495), PARP1 (9532), cleaved-PARP1 (5625), and SQSTM1/p62 (5114) were obtained from Cell-Signaling Technology (Danvers, MA, USA). ACTB/β-actin (A5441) 3-Methyladenine (M9281), and Chloroquine (C6628) were purchased from Sigma-Aldrich (St. Louis, MO, USA). LysoTracker Red DND-99 (L7528) was procured from Invitrogen. Bafilomycin (11038) was purchased from Cayman Chemical (Ann Arbor, MI, USA). DQ^TM^ Green BSA assay (D12050), Earle’s Balanced Salt Solution (EBSS; 14155-063), Alexa Fluor 488 (A-11008) and Alexa Fluor 555 (A-21428) were purchased from Thermo Fisher Scientific. (Waltham, MA, USA)

### 4.2. MTT Cell Viability Assay

The anchorage dependent viability of HCC cells after treatment with WFA alone and in combination with 3MA, CQ and Bafilomycin, respectively, was determined by estimating the reduction of MTT (3-(4,5-Dimethylthiazol-2-yl)-2,5-Diphenyltetrazolium Bromide) assay (Thermo Fisher Scientific, M6494) following manufacturer’s protocol. HCC cells were seeded at an initial density of 5 × 10^3^ cells/ well in 96 well plates for 24 h followed by treatment with various concentrations of WFA for 48 h. In a similar set of experiments, HCC cells were treated with a fixed concentration of WFA (5 µM), 3MA (4 mM), CQ (25 µM) and Bafilomycin (200 nM) alone and in combination (WFA+3MA, WFA+CQ and WFA+Baflo), respectively for 48 h. Then, 0.5 mg/mL MTT solution was supplemented to each well and incubated for 4 h at 37 °C for formazan crystal formation which was later dissolved in DMSO and absorbance was read at 570 nm using microplate reader (SPECTRAmax PLUS, Molecular Devices, Sunnyvale, CA, USA). The IC50 value was calculated using Microsoft excel and the combination index of multiple drug combinations was analyzed by Compusyn (Compusyn Inc., Paramus, NJ, USA) software.

### 4.3. Trypan Blue Dye Exclusion Assay

Trypan blue dye exclusion assay was performed to assess the number of viable cells after treatment with WFA alone or in presence of autophagic inhibitors. Viable cells resuspended in trypan blue dye were counted using hemocytometer, and the data is represented graphically.

### 4.4. Clonogenic Cell Survival Assay

Clonogenic cell survival assay was performed following our previously published protocol [57]. In brief, HCC cells were trypsinized and 500 cells per well were seeded in 12-well plates for 24 h followed by treatment with various concentrations of WFA. Every third day, the medium was replenished with fresh medium containing treatments. Following 10-day treatment period, the colonies were stained with crystal violet (0.1% in 20% methanol) and visually evaluated. Colonies having > 50 normal-appearing cells were counted. Pictures were taken using a digital camera.

### 4.5. Immunofluorescence Microscopy

HCC cells (2 × 10^5^ cells/well) were resuspended in eight-well chamber slides (Nunc, Rochester, NY, USA) and were allowed to adhere overnight. Then, the cells were treated with WFA and subjected to immunofluorescence analysis following the previously described protocol [44,58]. For the mRFP-EGFP-LC3B assay, hepatocellular carcinoma cells were seeded in eight-well chamber slides, transfected with mRFP-EGFP-LC3B (Addgene, 21074; deposited by Tamotsu Yoshimori) using Fugene (E2311, Promega, Madison, WI, USA) for 24 h followed by vehicle-control or WFA treatment. Upon completion of treatment, the cells were fixed with 4% paraformaldehyde in phosphate-buffered saline (PBS; 70-013-032, Fisher Scientific, Hampton, NH, USA) and fluorescence was analyzed microscopically. Cells containing GFP-LC3B+ puncta (green) or mRFP-LC3B+ (red) or GFP+ mRFP+ (yellow) puncta were examined and images were obtained using fluorescence microscope. All experiments were performed multiple times by using independent biologic replicates.

### 4.6. Immunohistochemical Staining

Immunohistochemical analysis for autophagy markers was performed as mentioned earlier [59]. Fixed and blocked slides were probed with primary antibodies followed by HRP conjugated secondary antibody. The slides were developed using DAB peroxidase substrate kit (SK-4100, Vector Laboratories, CA, USA). Images were captured in 20× magnification using bright field microscope (Zeiss, Axioplan, Germany).

### 4.7. Immunoblotting

Immunoblotting was performed following the protocol described earlier [60]. In brief, whole cell lysate and lysosomal extract were prepared using modified RIPA lysis buffer and lysosomal enrichment kit (Prod#89839, Thermo SCIENTIFIC, Waltham, MA, USA), respectively and equal amount of protein was resolved on sodium-dodecyl sulfate polyacrylamide gel. The proteins were transferred onto PVDF membrane and immunoblotted using specific antibodies.

### 4.8. Semi-Quantitative PCR

Semi-quantitative PCR was performed to analyze the mRNA expression pattern of autophagy related genes in vector-control or WFA treated HCC cells. Total RNA was isolated using TRIzol Reagent (Life Technologies Inc., Rockville, MD, USA). Gene specific amplification was performed using specific sense and antisense PCR primers.

### 4.9. Cathepsin-D Activity Assay

The lysosomal extracts of vehicle-treated and WFA-treated HCC cells were prepared as mentioned above. Equal amount of protein was used to measure the Cathepsin-D activity using Cathepsin D Activity Assay Kit (K143-100, BioVision, Milpitas, CA, USA) following manufacturer’s protocol.

### 4.10. TUNEL Assay for Apoptosis

Untreated and treated HCC cells were fixed in 4% paraformaldehyde and apoptotic cells were detected using In Situ Apoptosis Detection Kit (TACS^®^ 2 TdT DAB Kit, Trevigen, Gaithersburg, MD, USA) following the manufacturer’s instructions. TUNEL positive cells with dark brown nuclei were measured as a percentage of apoptotic nuclei versus total nuclei in three independent measurements. A dark brown DAB signal shows apoptotic nuclei, while blue-green to greenish tan specify a nonreactive cell.

### 4.11. Statistical Analysis

All the experiments were performed multiple times and the data represents independent biological replicates. Microsoft excel was used for statistical analysis of data analysis. Two tailed Student *t* test was used for measures of significance. *p* < 0.05 was considered to be significant.

## 5. Conclusions

In conclusion, our studies show that Withaferin A inhibits HCC growth and induces cytoprotective autophagy and simultaneous inhibition of cytoprotective autophagy increases the efficacy of Withaferin A in hepatocellular carcinoma. In this era of combination therapies to target heterogeneous tumors, our data presents evidence to support a combination therapeutic strategy involving WFA and autophagy inhibitors for HCC patients and warrants further studies.
